# Preliminary application of native Nephila edulis spider silk and fibrin implant causes granulomatous foreign body reaction in vivo in rat’s spinal cord

**DOI:** 10.1371/journal.pone.0264486

**Published:** 2022-03-14

**Authors:** Felix Koop, Sarah Strauß, Claas-Tido Peck, Thomas Aper, Mathias Wilhelmi, Christian Hartmann, Jan Hegermann, Julia Schipke, Peter M. Vogt, Vesna Bucan

**Affiliations:** 1 Department of Plastic, Aesthetic, Hand & Reconstructive Surgery, Hannover Medical School, Hannover, Germany; 2 Cardiac, Thoracic, and Vascular Surgery, Hannover Medical School, Hannover, Germany; 3 Department of Neuropathology, Hannover Medical School, Hannover, Germany; 4 Research Core Unit Electron Microscopy and Institute of Functional and Applied Anatomy, Hannover Medical School, Hannover, Germany; 5 Institute of Functional and Applied Anatomy, Hannover Medical School, Hannover, Germany; Dicle University: Dicle Universitesi, TURKEY

## Abstract

After spinal cord injury, gliomesenchymal scaring inhibits axonal regeneration as a physical barrier. In peripheral nerve injuries, native spider silk was shown to be an effective scaffold to facilitate axonal re-growth and nerve regeneration. This study tested a two-composite scaffold made of longitudinally oriented native spider silk containing a Haemocomplettan fibrin sheath to bridge lesions in the spinal cord and enhance axonal sprouting. In vitro cultivation of neuronal cells on spider silk and fibrin revealed no cytotoxicity of the scaffold components. When spinal cord tissue was cultured on spider silk that was reeled around a metal frame, migration of different cell types, including neurons and neural stem cells, was observed. The scaffold was implanted into spinal cord lesions of four Wistar rats to evaluate the physical stress caused on the animals and examine the bridging potential for axonal sprouting and spinal cord regeneration. However, the implantation in-vivo resulted in a granulomatous foreign body reaction. Spider silk might be responsible for the strong immune response. Thus, the immune response to native spider silk seems to be stronger in the central nervous system than it is known to be in the peripheral body complicating the application of native spider silk in spinal cord injury treatment.

## Introduction

According to the National Spinal Cord Injury Statistical Centre (NSCISC) 17,900 new cases of spinal cord injuries (SCI) are on record in the United States each year. These lead to a prevalence of approximately 296,000 persons affected in 2021 [1]. Functional limitations following SCI, which depend on the spinal level and the pathology of the damage, can be rigorous. Additionally, the psychological consequences can elevate depression and anxiety and decrease quality of life [2–4].

Dependent on the pathology of injury, the spinal cord can recover spontaneously (e.g., by axons bypassing the lesions, so-called sprouting) except total transection, which is known to be not able to regenerate spontaneously in humans and rats [5]. Even though spinal cord regeneration after complete transection has been described in various animal studies with diverse therapeutic strategies [6], there is still no convincing standard curative therapy available for humans. Current treatments focus on preventing secondary damage and rehabilitating the patient to the greatest possible independence [7, 8].

It is consensus that a curative therapy will most likely be a combinatorial approach built by, among others, neuroprotective and growth-stimulating substances, electrical stimulation, and a scaffold to bridge the defect [9–12]. One major problem after spinal cord injury is so-called glial scaring, hindering axonal regeneration as a physical barrier [13]. This can be excluded by the implantation of scaffolds serving as bridges. When it comes to the application of implants or transplants, autologous nerve tissue (if a tension-free end-to-end reconnection is not achievable) is the current gold standard for the treatment of peripheral nerve injuries [14], and it is also known to support regeneration after SCI [15]. However, the availability of autologous tissue is limited and associated with donor site morbidity. On the other hand, without immunosuppression, the implantation of allogenous nerve tissue is known to cause immunological problems such as rejection of the graft [16]. So, there is still a need for alternative natural or synthetic biomaterials which can be used to bridge these lesions and support axonal regeneration.

Due to remarkable progress in tissue engineering, various implants have been developed in recent years [17–19]. These scaffolds have to feature various characteristics. First, they need to be biocompatible, not causing significant inflammation. Secondary, biodegradability, as well as permeability, is indispensable. Furthermore, the graft should support cell adhesion and migration, guiding the cell regeneration in an axial direction. Additionally, the mechanical characteristics are in direct relation to a potential foreign-body reaction [20]. Many natural materials, such as collagen, hyaluronic acid, chitosan, gelatine, agarose, alginate, fibrin, and self-assembling peptides, meet these requirements in various approaches [17]. On the other side, synthetic materials’ customizability has led to attempts to treat the CNS with these alone or in combination with natural substances [17]. Nevertheless, none has led to a significant improvement in standard therapy of SCI patients yet.

Spider silk is an attractive material. Due to its biocompatibility, good biodegradability, low density, and unique mechanical characteristics, spider silk has been used in various medical approaches [21]. Among others, it was applied as a peripheral nerve graft in-vivo in sheep and rats, showing promising results: migration of various cell types, causing axonal outgrowth and remyelination [22–24]. Regarding the central nervous system, *Antheraea pernyi* silkworms’ filaments have been tested as a potential biomaterial for spinal cord injury therapy. Varone et al. induced an improved neuronal migration in vitro and only a minimal inflammatory response in-vivo when filaments were implanted under the dura mater of healthy rats [25]. Recombinant spider silk combined with neural precursor cells has been used to build a construct to improve regeneration in Rhesus macaque monkeys and suggested good biocompatibility [26].

To our knowledge, native spider silk has not been investigated for in-vivo application in the CNS yet. Due to the unique characteristics of native spider silk and positive results in peripheral nerve regeneration, it should be evaluated whether this material might also be helpful as a biological scaffold to bridge long-distance lesions and support neurite outgrowth after SCI.

For this study, the dragline silk of female *Nephila edulis*, also known as Australian golden orb weavers, was used. The species is easy to breed under laboratory conditions, and harvesting large silk quantities does not present a problem either [27]. While the industrial production of native spider silk seems possible, the advantages of recombinant silk cannot be denied here. Dragline silk is one of eight different silk types araneids produce for different intended purposes.

To prevent non-neuronal cells from migrating from the lateral direction and axially structure the silk, silk threads were combined with fibrin in a two-component construct. It consists of a condensed fibrin tubule, ensheathing native spider silk threads held in position by a liquid fibrin filling of the lumen. Fibrin alone already had been tested as a scaffold in the CNS, improving the sprouting of neural fibers and inhibiting reactive astrocytes that cause scarring [28]. Fibrin is very easy to form for the desired function. Therefore, it had also been used in various other tissue-engineering applications [29].

This study aimed at designing a spider silk-based scaffold to enhance regeneration after spinal cord injury. To align spider silk in longitudinal orientation, we combined it with a fibrin tunnel.

## Materials and methods

### Harvest of dragline silk

**Fig 1:**
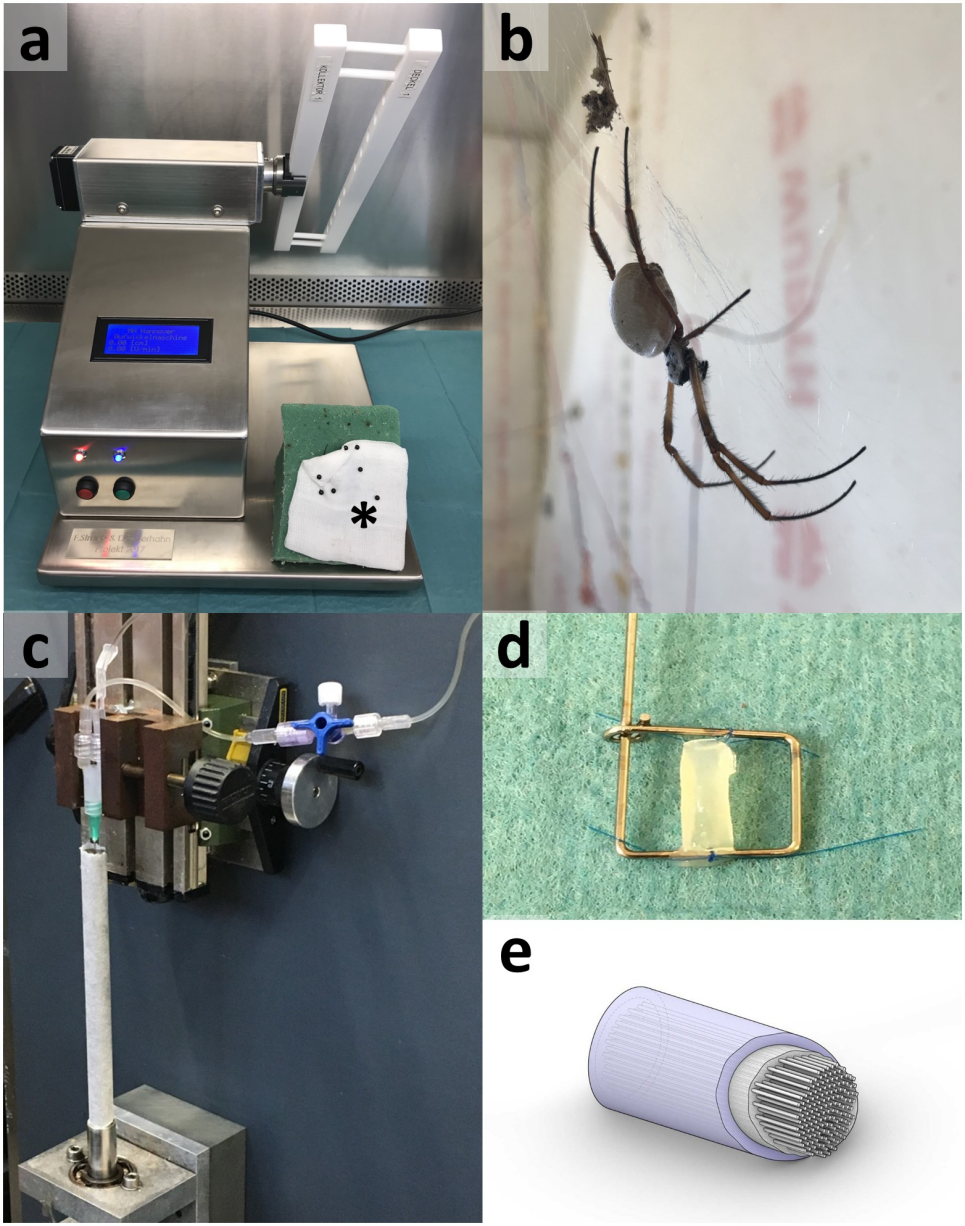
Construct fabrication. A: computerized reeling machine for spider silk harvest [30]. The spider is carefully fixed underneath a compress with needles placed between the spiders’ legs and in front of the head, indicated by a star. B shows a female *Nephila edulis* spider in the department’s animal facility. In c, the apparatus for fibrin manufacturing can be seen. D shows the fibrin sheath containing spider silk (reeled between the metal frame). E is a scheme of the implant. It shows the dense fibrin tube containing the longitudinally orientated spider silk which is held in position by a fibrin filling.

For all trials, native dragline silk of female *Nephila edulis* spiders (Fig 1b) of the department’s own keeping was used. Adult spiders were housed freely as previously described [27] in an own room with large windows and additional illumination at 25 to 30°C. Animals were watered daily and fed twice a week with adult or semi-adult crickets (*Achaeta domestica*).

For silk harvest, spiders were collected from their webs using clean plastic containers and fixed on a foam block. Using a small tweezer, dragline from the major-ampullate-gland was applied to a collector attached to the reeling machine [30] (Fig 4a). By winding of the collector, dragline was reeled. Spiders themselves do the same by using their hind legs. To treat the spiders with care, reeling was limited to 15 minutes per spider. Around 30 meters per animal were harvested. For silk collection, sterile gloves, surgical masks, and hairnets were worn to reduce contamination. For in vitro and in vivo trials, silk was autoclaved at 121°C for 20 minutes. After reeling, animals were placed back in their individual webs and rewarded (fed) with crickets.

### Manufacturing of fibrin tube

Fibrin tubes were manufactured as previously described [31]. A fibrinogen solution (Haemocomplettan^®^,CSL Behring, Marburg, Germany) was simultaneously applied with a thrombin-solution into a custom-made rotating mould (Fig 4c). The mould was a 10 cm long metal tube, inner diameter 9 mm, with a total of 14 drainages boreholes, 0.5 mm diameter, distributed pairwise and opposite over the circumference, with a Teflon inset of two half-moulds forming a polytetrafluoroethylene coating of the inner mould surface to an inner diameter of 7 mm. Fibrinogen containing mixture was assembled by concurrently introducing 10 mL aqueous fibrinogen solution at a flow rate of 120 mL/h having a concentration of 20 to 30 mg/mL fibrinogen with a thrombin solution containing 200 units thrombin (Baxter, Unterschleissheim, Germany) and 2,000 units aprotinin (Loxo, Dossenheim, Germany) dissolved in CaCl-solution (40 mmol/ml) at a flow rate of 80 mL/h, each to coupling leading to one common tubing that discharged into the mould while the mould was rotated at 5,500 rpm, resulting in a centrifugal force about 120 x g. The common tube was connected to a 0.9 x 40 mm cannula introduced axially in the mould and retracted continuously from the mould during application of the mixture of the fibrinogen soliton and the thrombin solution. Afterwards, the mould was rotated about its longitudinal axis with 10,000 rpm, resulting in a g-force of about 400 x g on the mould’s inner surface for about 20 minutes. Subsequently, the tube of polymerized fibrin was removed from the mould. Before drying, the sheath had a maximum tensile strength of 10 to 17 kPa and an elastic modulus of 0.8 to 1.5 kPa.

Compaction of the fibrin was done by a drying process and subsequent rehydration. Before drying the fibrin sheath at room temperature, a plastic cannula was inserted into the sheath to prevent it from collapsing. The dried sheath was then re-hydrated in aqueous solution aqua ad iniectabilia with 40 mmol CaCl_2_, containing 1% vol antibiotic solution (CellCultureGuard, PanReac Applichem) and 1,000 units/mL aprotinin. Re-hydration took one hour at 4°C, afterward the insert could be retracted, and then the sheath could be stored in this solution at 4°C. After drying and re-hydration, the fibrin sheath had a maximum wall tension, also referred to as tensile strength, that was increased compared to a fibrin sheath directly after polymerization in the mould and without drying. A maximum wall strength of 12.4 to 46.1 kPa was determined for the dried and re-hydrated fibrin sheaths from several repetitions. The pores between fibrin fibrils of the dried and re-hydrated fibrin sheath from several repetitions were determined to have a diameter of 13–80 μm.

### Construct fabrication

Sterile spider silk threads were carefully inserted into an 8 mm long fibrin tube. All in all, 100 to 200 fibers were arranged longitudinally in one tube (Fig 1d). Afterward, it was filled with a mixture of 20 mg/mL fibrinogen in aqua, admixed with ten units of thrombin in 40 nM CaCl_2_. The fibrin, filling the lumen of the tube and enclosing the silk fibers within, had pores between fibrin fibers of 164 ± 31 μm. A scheme of the implant is shown in Fig 1e.

### Culturing of OECs on spider silk and fibrin tunnel plus viability essay

Rat’s OECs had been isolated previously for other purposes [32]. OECs were cultivated on fibrin tube and spider silk matrices for three weeks in a medium mix of 50% modified Eagle’s medium (Cell Culture Company, Minneapolis, USA), 25% heat-inactivated horse serum (Sigma-Aldrich, St. Louis, USA), and 25% Earle’s Balanced Salt Solution (Life Technologies, Carlsbad, USA) supplemented with 36 mM D-Glucose (Sigma-Aldrich, St. Louis, USA) in a cell culture incubator at 37°C, 95% humidified air and 5% CO2. The cultivation medium was changed twice a week.

After three weeks, a viability assay was performed (LIVE/DEAD^®^ Viability/Cytotoxicity Kit for Mammalian Cells, ThermoFisher Scientific, Waltham, USA). According to the manufacturer’s protocol, the staining solution was composed with 5 μL calcein AM and 20 μL ethidium homodimer-1 (EthD-1) ad 10 mL Dulbecco’s phosphate-buffered saline (DPBS). Calcein accumulates in vital cells, causing green fluorescence (ex/em ~495 nm/~515 nm). EthD-1 binds to nucleic acids of dead cells. Here it turns out as a red fluorescence (ex/em ~495 nm/~635 nm). After the medium was removed from the cells, 100–200 μL of the staining solution were added and incubated for 30 minutes at room temperature. Using a fluorescein isothiocyanate (FITC) and a Red Fluorescent Protein (RFP) filter, samples were evaluated with a fluorescence microscope (CK40, Olympus, Tokio, Japan) and a ColorView 11 camera (Soft Imaging Systems GmbH, Münster, Germany).

### Ex vivo culture of spinal cord explants

Three donor animals were sacrificed according to the German Animal Welfare Act Guidelines. The spinal cord was extracted surgically and cut into pieces measuring 3 x 3 x 3 millimetres. Tissue pieces were washed once in Hanks’ Balanced Salt Solution (HBSS) (Thermo Fisher Scientific, Waltham, USA) before being placed on six stainless steel 1 cm^2^ wireframes reeled unidirectional with native spider silk. Before seeding, frames with silk had been autoclaved as described above. Explants were cultivated for four weeks using the medium and culture conditions described above. The medium was exchanged every two to three days. After four weeks, a part of the samples was fixed with 1,5% paraformaldehyde/ 1,5% GA HEPES (0,15 M) for 30 minutes and prepared for scanning electron microscopy (SEM). SEM was performed using a Zeiss Crossbeam 540-47-80 microscope. The other samples were prepared for immunofluorescence staining by fixation with 4% paraformaldehyde for 20 minutes. For fixation and permeabilization, Image-iT^™^ Fixation/Permeabilization Kit (ThermoFisher Scientific, Waltham, USA) was used. After fixation for 15 minutes in 4% paraformaldehyde, samples were washed three times in Phosphate-Buffered Saline (PBS) for 2–5 minutes. Afterward, cells were permeabilized using 0,2% Triton X-100 for 15 minutes. After repeated three times washing in PBS, samples were blocked for 60 minutes with 2% (w/v) Bovine Serum Albumin (BSA) in PBS. After three-times washing in PBS, first antibodies were applied with a dilution of 1:100 each in a 1% BSA/ PBS mixture overnight at 4 °C. As primary antibodies, anti-beta III tubulin antibody (rabbit anti-rat) (Abcam, Cambridge, United Kingdom), anti-nestin antibody (mouse anti-rat) (Abcam, Cambridge, United Kingdom), anti-myelin oligodendrocyte glycoprotein antibody (mouse anti-rat) (Abcam, Cambridge, United Kingdom), anti-CX3RC1 antibody (rabbit anti-rat) (Amsbio LLC, Abingdon, United Kingdom), and anti-glial fibrillary acid protein (rabbit anti-rat) (Agilent, Santa Clara, United States) were used. On the following day, samples were washed three times with PBS. Then secondary antibodies were applied diluted 1:1000 in 1% PBS/ BSA mixture and incubated for 30 minutes. For secondary antibodies goat anti-rabbit IgG (H+L) (Alexa Fluor^®^ Plus 488, Thermo Fisher Scientific, Waltham, USA) and goat anti-mouse IgG (H+L) (Alexa Fluor^®^ Plus 488, Thermo Fisher Scientific, Waltham, USA) were used. After washing three times, the samples were dried and mounted with Immu-Mount (Thermo Fisher Scientific, Waltham, USA). Control samples were prepared with the same steps without first antibodies to exclude unspecified binding of secondary antibodies. DAPI (4′,6-diamidino-2-phenylindole) (Thermo Fisher Scientific, Waltham, USA) was used for cell nuclei counterstaining. Samples were analysed with a Zeiss Axiovert 200M microscope and a Zeiss AxioCam MRm camera (Zeiss, Jena, Germany).

### Training of surgical procedure

The surgical procedure was initially trained on two cadavers of Wistar rats. The back skin of the target region was shaved. Animals were placed in a ventral position. Skin disinfection was performed with Softasept N using sterile swabs. On the thoracic level 10 to 11, the skin was incised with a scalpel, and tissue underneath prepared exposing the spine. It was tried to spare muscles as well as possible. The spinal canal was opened on the same spinal level by removing the dorsal vertebral arch. The dura mater was opened. The unveiled spinal cord was lifted with a spinal cord hook and then severed twice with an 8.5 cm Vannas-Tübingen Spring Scissors leaving a defect of 8 mm in length. It was tried to spare the blood vessels. Extracted tissue was harvested and fixed with 4% PFA as healthy control. Directly after preparation of the defect, sterile fibrin-spider silk constructs of appropriate size were implanted. Therefore, the spinal cord ends were carefully embedded into the ends of the tube, resulting in direct contact with the silk fibres. Dura mater was closed with 5–0 absorbable suture material. The spinal canal was closed using the raised vertebral arch. The skin incision was sutured using absorbable suture material.

### Surgical procedure

All animals were treated according to the guidelines of the German Animal Welfare Act. The trial had been authorized by the Lower Saxony District Government (file number: 18/3014). Additionally, all animal experiments, were carried out in strict compliance with the ARRIVE guidelines. Considering the physical and the psychological stress of the animals suffering paraplegia combined with this work’s proof of concept, the ethics committee of the Lower Saxony District Government approved 4 adult Wistar rats, weighing 280g to 300 g. Due to well-described remodelling processes with detailed cell biology, no control group was approved. Rats were kept in conventional EU Type 4 cages equipped with filter hoods and espen litter. Food and water were provided with high caloric 1324 TPF extrudate and autoclaved water ad libitum. Litter was exchanged twice a week. Housing temperature was kept continuously at 22°C ±0,5 with a humidity of 55±5%. Animals were subject to a 14/10 day-night rhythm. The animals are deliberately ordered at an early age to have enough time to adapt to their new environment (7 days) and the subsequent training for the walking analyses, which had been planned to be a subject of later investigations. All surgical interventions were carried out using isoflurane-oxygen inhalation anaesthesia. The anaesthesia was carried out with 5% isoflurane and a flow rate of 1L / minute in a knockdown box, followed by further inhalation via breathing mask (1.5%–2.5% isoflurane) and a flow rate of 1L / min. Sufficient depth of anaesthesia was verified by testing the righting reflex, the eyelid reflex, and the toe-pinch reflex. During surgery, the toe-pinch reflex was tested at regular intervals. Bepanthen eye ointment served as protection for the animals’ eyes. After checking the eyelid reflex, it was applied to the open eyes and spread carefully over the cornea by manually moving the eyelids. Additionally, to the inhalation anaesthesia, Carprofen, Butorphanol, and Lidocaine were used for analgesia. Carprofen (0,1ml/100g KGW) and Butorphanol (0,1ml/100g KGW) were injected subcutaneous in the target region. Before preparation of Dura mater, three drops of Lidocainhydrochlorid 2% were applied. After an exposure time of three minutes, the surgical procedure proceeded. Implants were transplanted according to the protocol described above. Postoperative the anesthetized animals, which were still lying prone on the heated plate, received a subcutaneous injection of Carprofen (0.1ml / 100g body weight) for analgesia, Baytril (0.04 ml / 100g body weight) for prevention of infection, and prewarmed Ringer/glucose (5% Glucose in 0.9% NaCl solution, s.c., 5 ml bilateral) as a depot in to avoid dehydration. A wound check with, if necessary, appropriate care and a check for indications of the termination criteria were carried out three times a day. For extended analgesia, rats received Carprofen s.c. 5 mg/kg body weight each day for seven days. Antibiosis was carried out with Baytril 2.5% injection for four days (for ten animals: 950 μl water for injection + 50 μl Baytril: 100 μl / 200 g rat). Three times per day, the tension of the bladder was checked, if necessary, emptying the bladder, checking the colour of the urine, weighting the animals twice a day, documenting abnormal behaviour (e.g., as a sign of pain) as well as evaluating the general condition of the animals using a modified score (Table 1).

**Table 1:** Modified score for the evaluation of animals wellbeing.

Level one, very active	strong and fast movements (of the upper body) combined with normal food uptake
Level two, active	strong movements (of the upper body), occasional pauses of movements, normal food uptake
Level three, less active	adequate reaction to environmental stimuli, more pauses of movements, decreasing food uptake combined with a low level of weight loss, which can be explained with the muscle loss of the lower extremities
Level four, decelerated	somnolent, strongly decreased food uptake with a loss of weight higher than 15% of the initial weight, signs of inflammation or automutilation
Level five, lethargic	no activity, no food uptake, vocalizations, loss of weight of over 20%, signs of inflammation or automutilation

No treatment was necessary for animals with a score of one or two. Level three combined with signs of pain indicated additional treatment with Carprofen for analgesia. In case of no improvement post analgesia for 24 hours or reaching level four or five of the score, animals would have been narcotized and sacrificed.

Animals were sacrificed at the end of the experiment under deep 5% isoflurane anaesthesia.

### Histology

Fixed tissue was watered with tap water, dehydrated in ascending ethanol (JT Baker, Phillipsburg, USA), and embedded in paraffin wax (Carl Roth, Karlsruhe, Germany). Samples were cut with an ultramicrotome (4 μm sections) (Leica Biosystems, Wetzlar, Germany). Afterward, standard histological staining, including haematoxylin and eosin (H&E) staining, iron staining (Prussian Blue), cresyl violet staining (Nissl staining), and elastic staining (Verhoeff Van Gieson / EVG) was performed. Slides were analysed with an Olympus BX46 (Olympus, Tokyo, Japan) microscope, and pictures recorded using an Olympus XC50 camera (Olympus, Tokyo, Japan). Additionally, immune fluorescence staining for GFAP and Tubulin was performed using antibodies and the protocol described above.

## Results

### Cultivation of olfactory ensheathing cells on spider silk and fibrin

**Fig 2:**
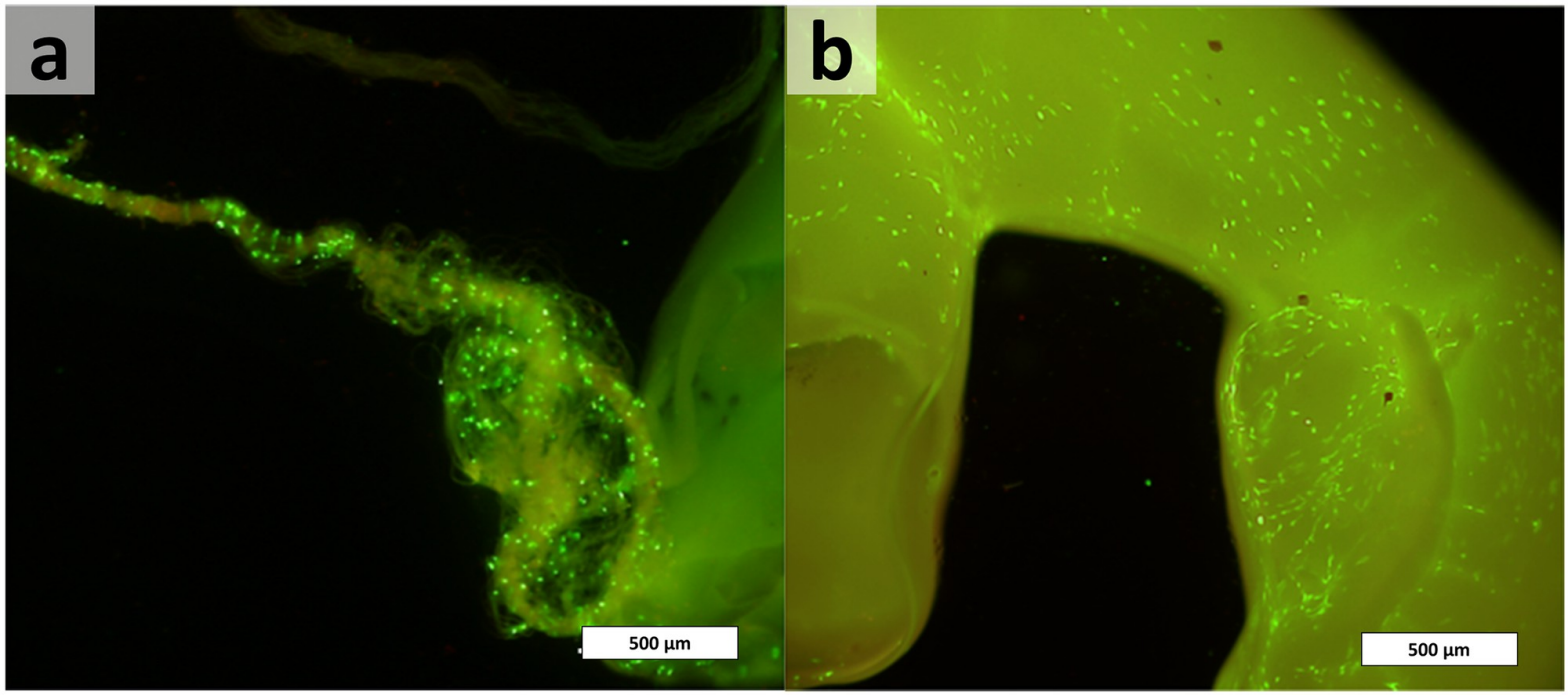
Life/dead assay. Spider silk (a) and fibrin scaffold (b) were seeded with olfactory ensheathing cells. Vital cells are green, while apoptotic cells (barely visible) appear red. Orange-red spider silk autofluorescence is faintly visible in a. Scale bars pertain to 500 μm.

As a first step, cells from the central nervous system seeded on spider silk and fibrin were evaluated. Therefore, olfactory ensheathing cells (OEC) from the cerebral cortex of a rat were isolated and seeded on spider silk and on the fibrin tunnel. After cultivation for 21 days, viability was proven by a double-staining fluorescence assay. In Fig 2a and 2b, the migration of OECs on and into the fibrin tube and the spider silk itself is visualized. Green cells are vital, while dead cells would appear red. There seems to be a high percentage of viable cells.

### Spinal cord tissue explants on spider silk

**Fig 3:**
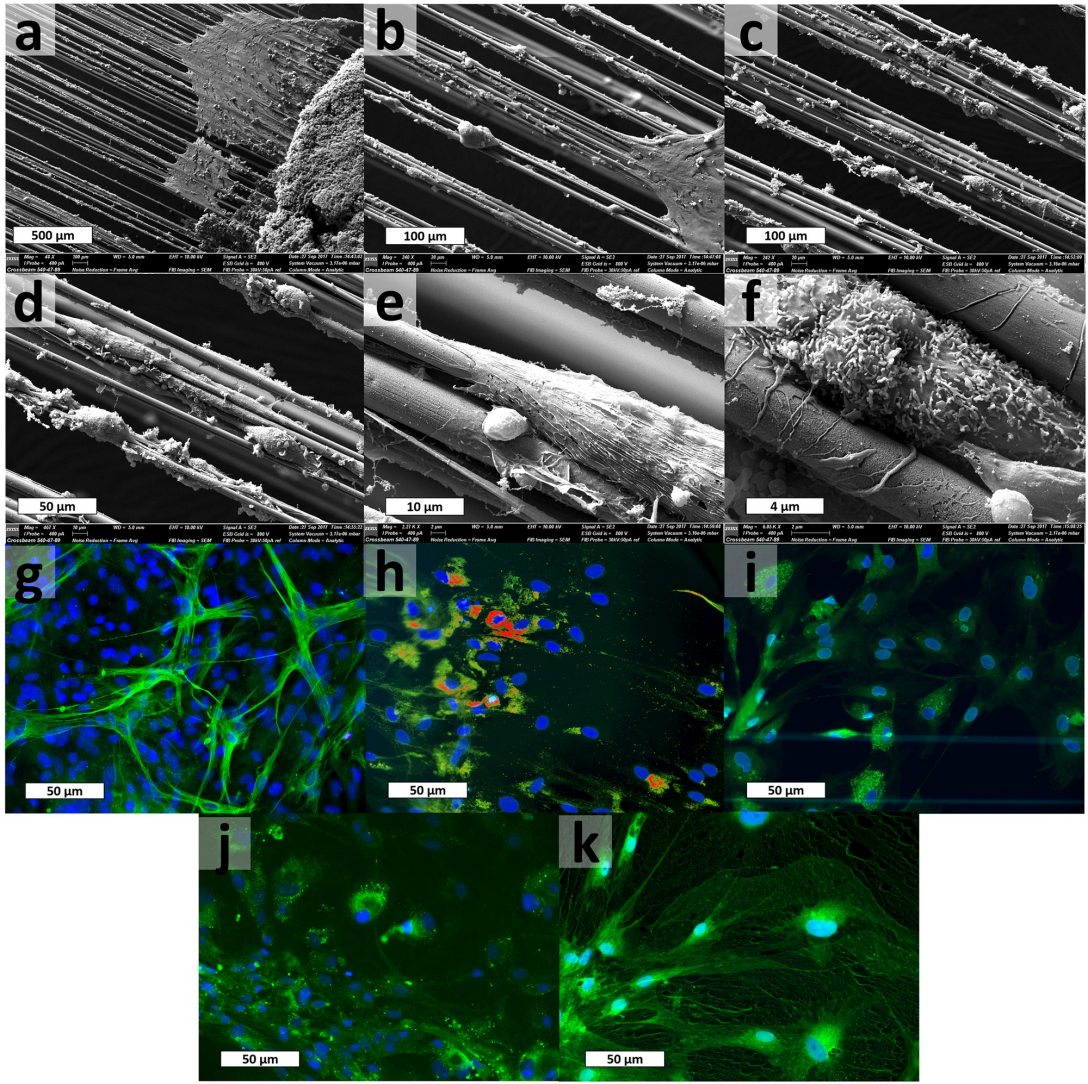
In-vitro examination of spinal cord tissue on spider silk. A-f Scanning electron microscopy of spinal cord tissue explant on spider silk after 21 days of cultivation. The tissue explant can be seen on the right site of a. Cells using the spider silk as guidance are documented in several magnifications. G-k Immuno-fluorescence staining of spinal cord tissue explant on spider silk after 21 days of cultivation. G Neuron-specific class III beta-tubulin (Tuj-1) indicating neuronal cell bodies and axons (green). H Nestin, intermediate filament protein, as a marker for neural stem cells (red). I CX3C chemokine receptor 1 (CX3CR1) indicating microglial-cells (green). J Myelin basic protein (MBP) indicating oligodendrocytes (green). K Glial fibrillary acidic protein (GFAP), intermediate filament protein indicating astrocytes and ependymal cells (green). Nuclei were counterstained with DAPI and are blue in all pictures.

Tissue explants originating from the spinal cord of three donor rats were prepared and placed on sterile spider silk scaffolds made of small metal frames reeled with silk. After three weeks of cultivation, the samples were examined using scanning electron microscopy and immune fluorescence staining. Scanning electron microscopy implies the motility of cells with several morphologies (Fig 3a–3f). The cells seem to build some kind of cluster or matrix (Fig 3a and 3b) over or by which they seem to move on the spider silk. By this, the neurites also seem to bridge gaps between individual fibres. These findings suggest that the cultivation of neuronal tissue explants on spider silk leads to the migration and adhesion of several cell types on the silk scaffold.

By immunofluorescence staining migrating cell types should be identified. The presence of neuronal cells bodies and their axons with beta-tubulin (Fig 3g), neuronal stem cells with nestin (Fig 3h), as well as different microglial cells (Fig 3i) like oligodendrocytes with myelin basic protein (Fig 3j) and astrocytes using the glial fibrillary acidic protein (Fig 3k) could be visualized.

### Spider silk-fibrin implant in vivo

**Fig 4:**
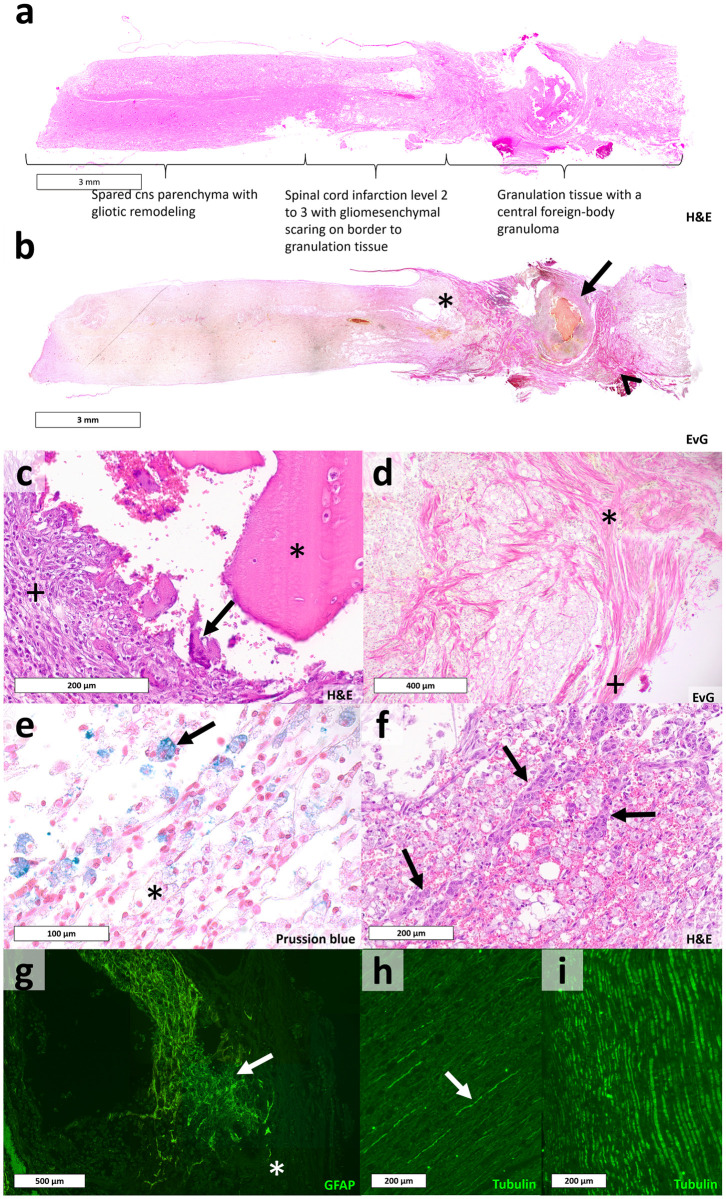
Foreign body reaction in-vivo. A (H&E) and b (EvG) display overview images of the spinal cord, which can be partitioned into three morphological sections. The foreign-body granuloma (b—arrow) and the migration of fibroblasts (b–arrowhead) are visible in b. In c (H&E) polynuclear foreign-body giant cells (c–arrow), epithelioid cells (c- plus), and the fibrin part of the initially placed implant (c–star) can be seen inside the foreign-body granuloma (b–arrow). D (EvG) is a magnification of b showing collagen (d–star) formed by migrating fibroblasts as a part of the gliomesenchymal scaring, starting from the arachnoid (d—plus). Hemosiderophages (e—arrow), indicating the old bleeding caused by the surgical procedure, and foamy macrophages (e–star) are visible in e (Prussion blue). Microvascular proliferation (f–arrows), as a part of the spinal cord infarction area, can be seen in f (H&E). G shows a magnification of the area marked in b with a star, stained for the glial fibrillary acidic protein, specifically expressed in astrocytes. No astrocytes are located beyond the transition to the implant (g–star). H and i were stained for tubulin, indicating neuronal bodies and axons. In h degenerated axons (h- arrow), in the form of Wallerian degeneration are visible, while i shows healthy axons, as a comparison, of the control.

The surgical procedure was trained on two rat cadavers. Afterward, the dragline silk-containing scaffold was implanted into four Wistar rats after complete transection and resection of the spinal cord of one centimeter. Animals’ wellbeing was controlled (uptake of water and food, weight, condition of the fur, behaviour) three times a day and evaluated using a specified score. Two animals did not recover well. Both showed decreasing uptake of food and lost weight too fast. According to the termination criteria, one rat was sacrificed after eight days and the second one on the 14th day. The remaining two rats tolerated the procedure considerably better and allowed an extension of the observation period from initially planned two to four weeks. However, during the short episode of 4 weeks, no motoric or sensitive improvements were observed. Histopathological analyses revealed a strong immune response causing a granulomatous encapsulating process due to a foreign body reaction in all sections, holding the implant. Examples of typical histological results are visible in Fig 4a and 4b are overview images of the injured spinal cord. Morphologically it is separated into three different sections. The spared but activated spinal cord tissue, the spinal cord infarction area, and the non-neural granulation tissue hosting the granuloma. On the left side, the spared reactive CNS parenchyma can be seen. It is characterized by hypertrophic reactive astrocytes and degenerated axons (Fig 4h- arrowhead) in the sense of Wallerian degeneration. Neuronal bodies are partly vital and partly degenerated. The same morphology was observed in the spared vital parenchyma at a higher thoracic level (not shown). The spared tissue develops into a spinal cord infarction area of level two to three. This is distinguished by a high concentration of foamy macrophages (Fig 4e–star), microvascular proliferation (Fig 4f- arrows), and reactivated microglial cells. The transition between the old spinal cord parenchyma and the non-neural granulation tissue can be seen in Fig 4g, which is in the star area in Fig 4b. Here the glial scar, built by activated astrocytes, limits the CNS parenchyma and borders the granulation tissue. The granulation tissue is formed by fibroblasts, migrating from the arachnoid (Fig 4d–plus), forming collagen, microvascular proliferation, and foamy macrophages. The accumulation of fibroblasts and collagen (Fig 4b–arrowhead) increases to the foreign-body granuloma (Fig 4b–arrow). The foreign-body response is indicated by the presence of polynuclear giant cells (Fig 4c- arrow). The granuloma, encapsulating the fibrin scaffold (Fig 4c- star), is also built by epithelioid cells (Fig 4c- plus). The sequence with the spinal cord infarction bordered by spared parenchyma would be expected to follow to the right side but was lost during preparation. Such a pathology excludes the possibility of a reorganization of functioning axons beyond the defect. Accordingly, the fluorescence staining against tubulin, a crucial protein in neuronal cell bodies and axons, also showed degenerated axons in Fig 4h in contrast to a control (Fig 4i). Furthermore, there were no significant amounts of granulocytes in the area of the granuloma, so that a septic inflammatory reaction as a possible consequence of pathogens entering the implant seems to be excluded.

## Discussion

After spinal cord injury, astrocyte scarring hinders axonal regeneration by building a physical barrier. The implantation of scaffolds targets on bridging this defect. Many different materials have been tested for this approach, while none has improved standard therapy. Spider silk is known for its good biocompatibility and has been tested in various medical applications [21]. In contrast to native silkworm silk of *Bombyx mori*, which in common belief induces a stronger immune response due to the coating protein sericin, spider silk showed no or only mild immune responses when applied in a medical setting [22–24]. Additionally, native spider silk was shown to improve axonal regeneration and remyelination in extended nerve defects in sheep [23]. However, native spider silk has not been tested in the CNS yet. Closest to it comes applying *Antheraea pernyi* silkworm silk under the dura mater of a healthy rat [25] or recombinant spidroin as a carrier for neural precursor cells into the brain and onto the spinal cord of healthy rhesus macaque monkeys [26]. While none of these experiments showed a foreign body response or significant inflammation, both treated healthy animals and did not set a lesion in which the implant was then placed. Therefore, this work aimed to design a native spider silk-based implant for improved regeneration after spinal cord transection. A fibrin tube was manufactured to align the spider silk longitudinally. In vitro, the fibrin tubes and the spider silk’s biocompatibility were investigated. Here a live/ dead assay was used, showing a high percentage of viable cells and suggesting no or only minor cytotoxicity. These results are in line with other publications, reporting no or only minor cytotoxicity of spider silk in various in vitro settings [33, 34].

Due to those findings, tissue explants originating from rat’s spinal cord were placed on spider silk. After three weeks of cultivation, scanning electron microscopy implied the motility of cells with several morphologies. The cells seem to build a cluster or matrix over or by which they move forward on the spider silk. Here, the neurites seem to bridge gaps between individual fibres. These results suggest that the cultivation of neuronal tissue explants on spider silk leads to migration and adhesion of several cell types on the silk scaffold. These findings are also supported by the literature [35, 36], where the migration and adhesion of neuronal cell types are well described. Additionally, the presence of neuronal cells bodies and their axons, neuronal stem cells, and microglial cells like oligodendrocytes and astrocytes were visualized using immunofluorescence staining.

An initial proof of concept model was set up with four rats. While the surgical procedure seemed to be tolerated well by two animals, allowing an extension of the experimental duration, the histological analyses revealed a significant immune response in the sense of a granulomatous foreign body reaction. Taking results of other studies [23–26] and the in vitro findings of this study into account, this was not expected in the present intensity. There are several reasons, possibly explaining a foreign body response in the spinal cord. Regarding the fibrin sheath, an implant should meet the mechanical characteristics of the host tissue. This can be explained with the glial cell mechanosensitivity, which can induce an immune response in the CNS [20]. Therefore, the elastic modulus of an implant should match the elasticity of the host tissue as well as possible. Depending on the measuring methods, the physiological elastic modulus of the rat’s spinal cord is described with around 100 Pa [37]. The implanted scaffold had an elastic modulus of 800 to 1.500 Pa. The resulting stiffness mismatch might be able to induce an immune response.

Evaluating this possibility, the fibrin used in other approaches in the spinal cord [38] was compared: Some researchers used fibrin glue (e.g., Baxter TISSEEL) [39]. While the safety of fibrin glue was proven and suggested no increased inflammation compared to the control group when treating spinal cord injuries [40], the elastic moduli remain with 102 +/- 41 kPa [41], remarkably higher than in our application. Other approaches, including ours, used fibrinogen and thrombin solutions, creating fibrin [42]. There is no information regarding the elastic moduli of the implants used by other groups, but since they used similar methods, it might be assumed that the characteristics might be related. While the mechanical characteristics of the fibrin sheath used here might not have met the demanded qualities, it is unlikely that fibrin caused such a strong immune response due to its mechanical characteristic. However, a softer biomaterial might show better acceptance. Another obstacle regarding the foreign body reaction could be the origin of the fibrin. Human thrombin and fibrinogen solutions were used for the manufacturing of the fibrin tube. More specific, Haemocomplettan was applied which contains human albumin. Nevertheless, most other approaches with fibrin in the spinal cord also applied human components [38–40] and did not report a foreign body reaction when used in rats. For example, Baxter Tisseel is commonly used and also contains human albumin. Therefore, the origin of fibrin might be of secondary importance when it comes to triggering an immune response. The use of allogenic or even autologous fibrin could still reduce the risk of non-homologous or xenogeneic immune rejections. Additionally, purified fibrin gained from fresh frozen plasma might reduce the risk of foreign body reaction.

Regarding the mentioned application of *Bombyx mori* silk [25] and recombinant spidroin [26], none of these experiments reported a foreign body response or major inflammation. However, in both trials, the material was implanted atraumatic without causing a lesion of the CNS parenchyma. This is important to mention since the physiological wound response is known to be imitated by the foreign body response in the CNS [43]. Therefore, a foreign body response is expected to be more severe in a traumatic scenario, given the same cellular initiation. Consequently, neither subcutaneous implantation nor in vitro examination guarantee a good in vivo biocompatibility when applied in traumatized CNS parenchyma. After spinal cord damage, the affected tissue gets isolated by multicellular interactions [44]. This isolation allows recruited inflammatory cells to resolve potentially toxic necrotic tissue while protecting spared vital tissue. Here, microglia and astrocytes, brain-resident immune cells, are early responders with limited capability of phagocytosis. By their expression of cytokines and chemokines, blood-borne immune cells are recruited to contribute to the degradation and phagocytosis of potentially noxious elements, like necrotic tissue or an as foreign recognized biomaterial. Additionally, perivascular mesenchymal cells and fibroblasts, migrating from the arachnoid, proliferate into the lesion, causing glio-mesenchymal scarring. While the implantation of scaffolds targets bridging the physical barrier to enable axonal regeneration, implantation of the fibrin spider silk scaffold caused blood-borne inflammatory cells to persist and cause a granulomatous encapsulation. This indicates a foreign body reaction caused by the implant. However, the foreign body response seems to be initiated by the same multicellular reactions like physiological wound responses in the CNS [43] and, therefore, does not occur in the peripheral tissue, explaining good biocompatibility of implants in the peripheral body. Since spider silk has not been applied in injured CNS, in contrast to fibrin, it is reasonable that native spider silk induced this multicellular CNS-specific wound response, causing the severe foreign body reaction observed histopathologically. However, the hypothesis that the observed reaction was due to differences between CNS wound response and that in peripheral tissue cannot be proofed by the present study and requires further examination. Nevertheless, the very same spider silk has been used in different in-vivo experiments so far [22–24] and did not show foreign body reactions. Alternatively, to dragline silk alone, the combination with fibrin or the implantation technique could have led to such a response. Still, the proof of concept resulted in foreign-body reactions, which are likely to be caused by the implantation of native spider silk. While the biocompatibility of an implant is crucial, the performance of the implant could be improved with antiapoptotic substances [10, 45]. Nevertheless, further tests are needed until native spider silk should be evaluated in a regenerative setting in the spinal cord again. The observed foreign body reaction does not require further examination. In case of a more positive outcome, the histological results shall be examined in a more standardized way using quantitative morphometric analysis with immunochemistry images [46]. Also, the hypothesis that the acceptance of a material regarding an immune response depends on the level of traumatization during the implantation requires further research.
